# EGF receptor modulates HEV entry in human hepatocytes

**DOI:** 10.1097/HEP.0000000000000308

**Published:** 2023-02-07

**Authors:** Jil A. Schrader, Thomas L. Burkard, Yannick Brüggemann, André Gömer, Toni L. Meister, Rebecca M. Fu, Ann-Kathrin Mehnert, Viet L. Dao Thi, Patrick Behrendt, David Durantel, Ruth Broering, Florian W. R. Vondran, Daniel Todt, Volker Kinast, Eike Steinmann

**Affiliations:** 1 Department for Molecular and Medical Virology, Ruhr University Bochum, Bochum, Germany; 2 Department of Infectious Diseases and Virology, Heidelberg University Hospital, Cluster of Excellence CellNetworks, Heidelberg, Germany; 3 Heidelberg Biosciences International Graduate School, Heidelberg University, Heidelberg, Germany; 4 German Center for Infection Research (DZIF), Partner Site Heidelberg, Heidelberg, Germany; 5 TWINCORE Center for Experimental and Clinical Infection Research, a Joint Venture between the Hannover Medical School (MHH) and the Helmholtz Center for Infection Research (HZI), Institute for Experimental Virology, Hannover, Germany; 6 Department of Gastroenterology, Hepatology and Endocrinology, Hannover Medical School, Hannover, Germany; 7 German Center for Infection Research (DZIF), Partner Site Hannover - Braunschweig, Hannover, Germany; 8 CIRI—International Center for Infectiology Research, Univ Lyon, Université Claude Bernard Lyon 1, Inserm, U1111, CNRS, UMR5308, ENS Lyon, Lyon, France.; 9 Department of Gastroenterology, Hepatology and Transplant Medicine, University Hospital Essen, University Duisburg-Essen, Essen, Germany; 10 Department of General, Visceral and Transplant Surgery, Hannover Medical School, Hannover, Germany; 11 European Virus Bioinformatics Center (EVBC), Jena, Germany; 12 Department of Medical Microbiology and Virology, Carl von Ossietzky University Oldenburg, Oldenburg, Germany; 13 German Center for Infection Research (DZIF), External Partner Site, Bochum, Germany

## Abstract

Being the most common cause of acute viral hepatitis with >20 million cases per year and 70,000 deaths annually, HEV presents a long-neglected and underinvestigated health burden. Although the entry process of viral particles is an attractive target for pharmacological intervention, druggable host factors to restrict HEV entry have not been identified so far.

**Approach and Results:**

Here we identify the EGF receptor (EGFR) as a novel host factor for HEV and reveal the significance of EGFR for the HEV entry process. By utilizing RNAi, chemical modulation with Food and Drug Administration–approved drugs, and ectopic expression of EGFR, we revealed that EGFR is critical for HEV infection without affecting HEV RNA replication or assembly of progeny virus. We further unveiled that EGFR itself and its ligand-binding domain, rather than its signaling function, is responsible for the proviral effect. Modulation of EGF expression in HepaRG cells and primary human hepatocytes affected HEV infection.

**Conclusions:**

Taken together, our study provides novel insights into the life cycle of HEV and identified EGFR as a possible target for future antiviral strategies against HEV.

## Graphical abstract

**Figure:**
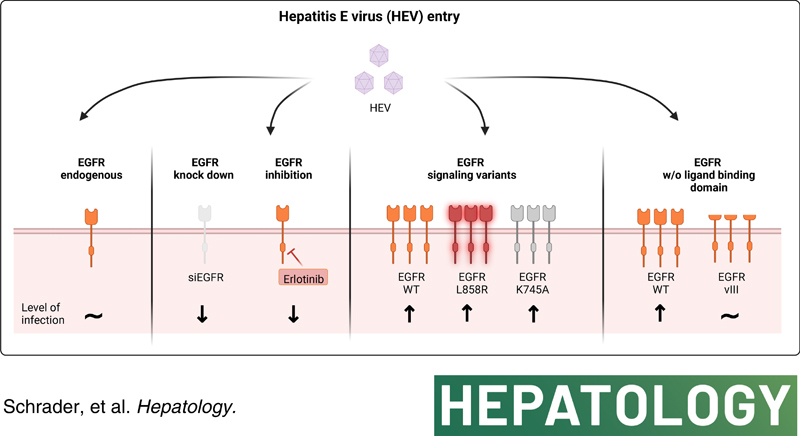

## INTRODUCTION

HEV is the main cause of acute viral hepatitis, creating endemic, waterborne outbreaks in developing countries and increasing zoonotic danger in the developed world, with estimated 20 million infections, leading to 3.3 million symptomatic cases and 44,000–70,000 deaths per year.^1^ Clinical manifestations of HEV infections range from mild symptoms of acute hepatitis to fulminant hepatitis and chronic infections in immunocompromised patients, along with a fatality rate of up to 30% in pregnant women.^2^,^3^ In Europe, known cases of HEV infections have increased 10-fold from 2005 until 2015, with some countries even reporting exceeding cases of HEV than HAV, indicating that further studies into HEV’s pathophysiology are urgently needed.^4^ HEV is a quasi-enveloped, positive sense, single-stranded (ss+) RNA virus of the genus *Paslahepevirus* within the family of Hepeviridae.^5^–^7^ HEV’s icosahedral, 27–34 nm virion encapsulates a 7.2 kb RNA, which consists of 3 open reading frames (ORFs 1–3) encoding the viral replicase, viral capsid and a protein for which multiple functions have been described, respectively.^6^,^8^,^9^ Since a robust cell culture system has been developed only recently,^10^ little is known about molecular determinants involved in HEV’s entry and life cycle progression.^11^–^13^ First evidence suggests that, as its first attachment, the HEV ORF2 capsid protein interacts with heparan sulfate proteoglycans on the cell surface.^14^ After binding to a yet unknown receptor, the virion is endocytosed in a clathrin-dependent manner.^14^,^15^ Integrin-α3 has recently been discovered as a putative host factor for the entry of nonenveloped HEV particles.^16^ However, the role of integrin-α3 for the HEV entry is still poorly understood, and additional host factors may be required for HEV entry into the host cell.

The EGF receptor (EGFR) has been demonstrated to be critical for the entry of a number of hepatotropic viruses, including HBV and HCV.^17^,^18^ EGFR is a receptor tyrosine kinase of the ErbB family, which controls cell proliferation, migration, and differentiation.^19^ Upon ligand binding to the receptor’s extracellular ligand-binding domain, EGFR forms asymmetric homodimers and heterodimers with other ErbB family members in which one kinase domain brings the other kinase domain into an active state catalyzing autophosphorylation of multiple tyrosine residues in the C-terminal domain.^20^–^22^ Consequently, binding sites for adapter proteins are created, initiating the downstream signaling cascade that contains a network of around 200 proteins relaying on the extracellular signal inside the cell.^23^ After ligand binding, EGFR is internalized by either clathrin-dependent or clathrin-independent endocytosis to endosomes and either routed for degradation or recycling.^24^–^26^ Several mechanisms by which EGFR can facilitate numerous different viral infections have been described so far, including enabling viral entry into host cells.^26^,^27^ In the context of HCV infections, EGFR acts as a cofactor regulating interactions between the entry receptors CD81 and claudin-1 and the subsequent fusion with host cell membranes in a clathrin-dependent manner.^28^

In this study, we examined the role of EGFR and its signaling during HEV infection. Through siRNA-mediated knockdown of EGFR, chemical inhibition and modulation of EGFR by Food and Drug Administration–approved drugs, and ectopic expression of the receptor, we identified EGFR as a novel HEV host factor required during viral entry. In addition, the ability of EGFR modulators to effectively suppress HEV infection in authentic cell culture models such as HepaRG cells and notably primary human hepatocytes suggested that EGFR also plays a key role during HEV infection. Taken together, our study provides novel insights into the life cycle of HEV and identifies a possible target for future antiviral strategies against HEV.

## METHODS

### Cell culture

The human hepatoma cell line HepG2 (ATCC-Nr.: HB-8065) and 293T cells (ATCC-Nr.: CRL-3216) were cultured in DMEM-high-glucose (Gibco, Cat.Nr. 11965), supplemented with 10% (v/v) fetal calf serum (FCS, Capricorn, Lot.Nr. CPC21-4114), 1% (v/v) nonessential amino acids (Gibco, Cat.Nr. 11140050), 100 IU/mL penicillin, 100 µg/mL streptomycin (Gibco, Cat.Nr. 15140), and 2 mM l-glutamine (Gibco, Cat.Nr. 25030). For virus titration, a HepG2-subclone (HepG2/C3A) was used because of its greater infection efficiencies and cultured in Eagle’s minimum essential medium (Gibco, Cat.Nr.11095), supplemented with 10% (v/v) ultralow IgG-FCS (Gibco, Cat.Nr. 16250-078, Lot 1939770), 100 μg/mL gentamicin (Gibco, Cat.Nr. 15710), 2 mM l-glutamine, 1 mM sodium pyruvate (Gibco, Cat.Nr. 11360), and 1% (v/v) nonessential amino acids. HepG2 and HepG2/C3A cells were grown on rat collagen-coated (SERVA Electrophoresis, Cat.Nr. 47256.01) cell culture dishes. As described,^29^ undifferentiated HepaRG cells were cultured in HepaRG growth medium consisting of William Medium E (Gibco, Cat.Nr. 22551), supplemented with 10% (v/v) FCS, 100 IU/mL penicillin, 100 µg/mL streptomycin, 100 μg/mL gentamicin, 2 mM l-alanyl-l-glutamine dipeptide (GlutaMax, Gibco, Cat.Nr. 35050), 5 µg/mL insulin (Sigma-Aldrich, Cat.Nr. I9278), and 50 mM hydrocortisone hemisuccinate (Sigma-Aldrich, Cat.Nr. 1319002). For differentiation, 5×10^4^ HepaRG cells were seeded on 24-well plates and incubated for 14 days, followed by 14 days incubation in HepaRG growth medium, supplemented with 1.8% (v/v) DMSO Hybri-Max (Sigma-Aldrich, Cat.Nr. D2650). The medium was changed twice a week. Primary human hepatocytes (PHH) were prepared from nontumorous tissue obtained from freshly resected livers, as described.^30^,^31^ Written informed consent was obtained from all patients, and the study was approved by the Institutional Review Board (Ethics Committee) of the medical faculty at the University of Duisburg-Essen. All research was conducted in accordance with both the Declarations of Helsinki and Istanbul. Human biological samples were provided by the Westdeutsche Biobank Essen (WBE, University Hospital Essen, University of Duisburg-Essen, Essen, Germany; approval 18-WBE-048). PHHs were seeded into collagen I-coated culture plates and cultured in William Medium E, supplemented with 5% (v/v) FCS, 1% (v/v) nonessential amino acids, 100 IU/mL penicillin, 100 µg/mL streptomycin, 2 mM GlutaMAX, 2% (v/v) DMSO, 10 mM HEPES (Gibco, Cat.Nr. 15630), 5.4 µM hydrocortisone hemisuccinate, 5.5 ng/mL EGF (human, Med Chem Express, HY-P7109), and 5 µg/mL insulin. All cells were kept at 37 °C in a 5% (v/v) CO_2_ incubator.

All materials and methods describing virus production and all assays utilized in this study are specified in the Supplemental Information (http://links.lww.com/HEP/C666).

## RESULTS

### EGFR is abundantly expressed in human hepatocytes

Numerous hepatotropic viruses, such as HBV and HCV, have been shown to exploit the EGFR during the virus entry process.^17^,^18^ As very little is known about the host factors for HEV, we aimed to investigate the role of EGFR and its signaling during HEV infections.

**FIGURE 1:**
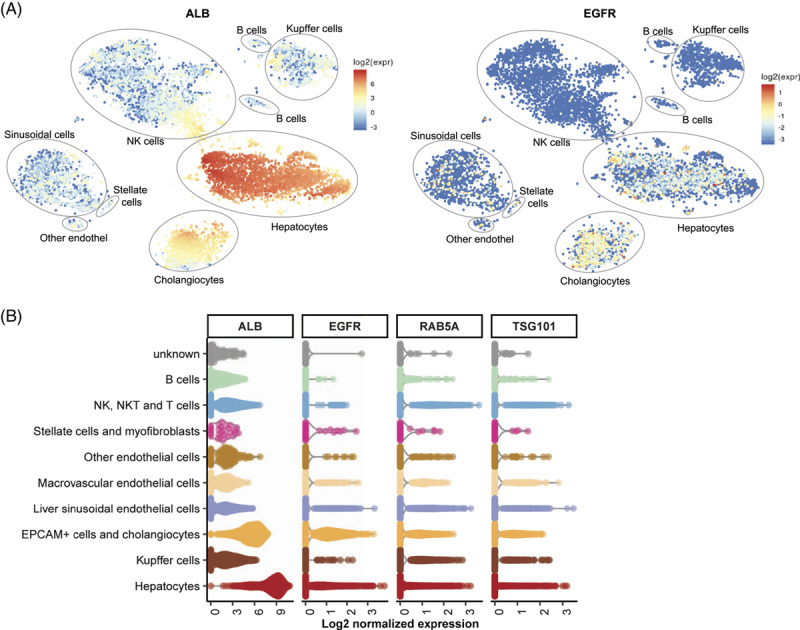
EGFR is expressed endogenously in primary human liver cells. (A) T-distributed stochastic neighbor-embedding (t-SNE) plots highlighting mRNA expression of ALB and EGFR across all cells of healthy human liver tissue.^32^ The color of each cell represents the gene expression, according to the corresponding legend as log2 value of the expression. Cell type annotation was transferred from Aizarani et al.^32^ (B) Violin plot showing the normalized expression of proposed HEV host factors EGFR, TSG101, and Rab5. The color code depicts the different cell types present in the data set. Abbreviations: EGFR, EGF receptor; NK, natural killer.

To determine the expression of EGFR in hepatocytes, we analyzed single-cell RNA-sequencing data of the human liver cell atlas from nine healthy human donors.^32^ T-distributed-stochastic-neighbor-embedding (t-SNE) plots highlighted the mRNA expression of the hepatocyte marker albumin (Figure 1A). Similar to the reported HEV host factors, TSG101 and Rab5, EGFR was highly abundant in clusters of hepatocytes and cholangiocytes (Figure 1B).

### Endogenous EGFR is critical for HEV infection

**FIGURE 2:**
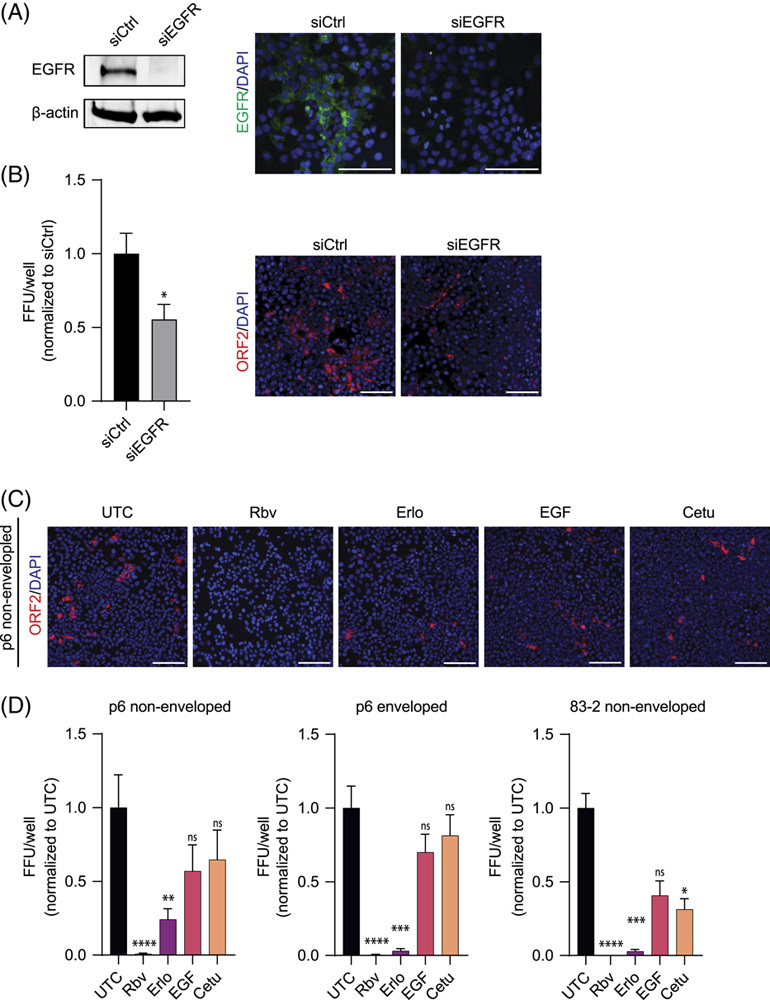
Endogenous EGFR is critical for HEV infection. (A) EGFR protein expression in HepG2 cells 48 h after transfection with EGFR-specific siRNAs or nontargeting control siRNA (siCtrl) analyzed by western blot (left) and immunofluorescence (right). (B) HEVcc (p6) infection in HepG2 cells transfected with EGFR-specific siRNAs and control siRNA. Cells were infected with HEVcc (p6) 2 d after transfection and FFU/well determined after fixation 5 d post infection. Left: quantification of FFU of the full well normalized to cells transfected with control siRNA. Right: representative immunofluorescence images stained for ORF2 protein. (C, D) HEVcc p6 nonenveloped and enveloped, as well as 83-2 nonenveloped infection in HepG2 cells under treatment of EGFR modulators erlotinib (33 µM, Erlo), EGF (16.5 nM), and cetuximab (34 nM, Cetu) compared with UTCs, whereas the HEV inhibitor ribavirin (50 µM, Rbv) served as control. (C) Representative immunofluorescence images stained for ORF2 protein in HEVcc (p6) infected HepG2 cells under EGFR modulator treatment. (D) FFUs/well were counted in HEVcc p6 nonenveloped (left), p6 enveloped (middle), or 83-2 (right) infected HepG2 cells under EGFR modulator treatment and normalized to UTC. To test the significance of mean differences, Student *t* test (B) and one-way ANOVA, followed by Dunnett multiple comparison test (D), were used. *p*-values <0.05 (*), <0.01 (**), <0.001 (***), and <0.0001 (****). *p*-values >0.05 were considered to be not significant. All infection experiments were performed in triplicates. Mean and SEM are depicted from at least three independent experiments. Scale bars = 100 µm. Abbreviations: EGFR, EGF receptor; FFU, focus forming units; ns, nonsignificant; ORF2, open reading frame 2; UTC, untreated control cells.

Given that hepatocytes are the main target of HEV during infection, we aimed to address the role of endogenous EGFR on HEV infection. Therefore, we performed siRNA-mediated knockdown, followed by HEV infection in HepG2 cells. The reduction of EGFR protein expression was confirmed by western blot and immunofluorescence staining (Figure 2A). Next, we infected EGFR knockdown and controlled HepG2 cells with cell culture-derived HEV (HEVcc) (p6), followed by immunofluorescence and focus forming units (FFUs) determination. We observed that silencing of EGFR reduced the number of HEV infection events by ~50%, demonstrating the significance of endogenous EGFR during HEV infection (Figure 2B). To confirm this data in a more authentic cell culture system, we used induced pluripotent stem cell (iPSC)-derived hepatocyte-like cells (HLCs) and transduced these with short hairpin RNA targeting EGFR (shEGFR), followed by infection with HEVcc (p6). The silencing of EGFR through shEGFR in HLCs was confirmed by western blot (Supplemental Figure S1A, http://links.lww.com/HEP/C666). The ratio of ORF2 protein positive (ORF2+) and transduced cells was lowered in shEGFR-transduced cells compared with shCtrl cells (Supplemental Figure S1B–C, http://links.lww.com/HEP/C666), confirming the crucial role of endogenous EGFR during HEV infection.

EGFR can be inhibited and modulated by multiple Food and Drug Administration-approved drugs, including erlotinib (Erlo), an EGFR-specific tyrosine kinase inhibitor, as well as cetuximab (Cetu), an antibody that competitively binds to the extracellular receptor domain and hinders receptor dimerization, thus impeding signal transduction. We aimed to explore the potential of these molecules as antivirals to combat HEV infection. First, we confirmed the ability of Erlo and Cetu to inhibit EGFR phosphorylation at the concentrations used in the following assays by immunofluorescence analysis of pEGFR(-1068) in serum-starved, EGFR modulator-treated HepG2 cells (Supplemental Figure S2, http://links.lww.com/HEP/C666). EGF, the cognate ligand of EGFR, served as a positive control to induce EGFR phosphorylation (Supplemental Figure S2, http://links.lww.com/HEP/C666). Subsequently, we infected hepatoblastoma cells with nonenveloped HEVcc of different strains (p6 and 83-2) and enveloped HEVcc (p6) in the presence or absence of different EGFR-specific modulators for the whole time of infection (5 d) (Figure 2C–D and Supplemental Figure S3, http://links.lww.com/HEP/C666). Treatment with the Food and Drug Administration-approved, EGFR-specific tyrosine kinase inhibitor, Erlo, resulted in a reduction of HEV p6, 83-2 and enveloped p6 infection events by ~76%, 99%, and 97%, respectively. Also, EGF, as well as Cetu, reduced HEV infection by 43% and 35% (p6), 59% and 69% (83-2), and 70% and 81% (enveloped p6), respectively. Of note, cell viability determined through 3-(4,5-dimethylthiazol-2-yl)-2,5-diphenyltetrazolium bromide assay was not affected by the applied concentration of the different EGFR modulators. In addition, Erlo was capable of inhibiting HEVcc (p6) infections in a dose-dependent manner (Supplemental Figure S4, http://links.lww.com/HEP/C666). These data suggest that the perturbation of endogenous EGFR can prevent HEV infection.

### Endogenous EGFR is required during HEV entry

To dissect which step of the HEV life cycle is affected by inhibition of endogenous EGFR, we performed different virological assays evaluating HEV attachment, entry, postattachment, replication, and assembly of progeny viruses in the presence or absence of EGFR modulators.

**FIGURE 3:**
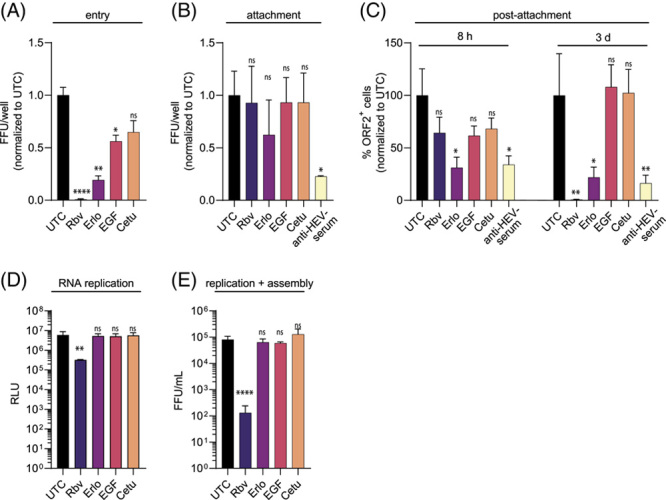
EGFR has no effect on attachment, replication, or assembly but affects the entry process of HEV. (A) Quantification of HEVcc (p6) entry in HepG2 cells under EGFR modulator treatment [erlotinib (33 µM, Erlo), EGF (16.5 nM), and cetuximab (34 nM, Cetu] compared with UTC. Cells were pretreated for 30 min with EGFR modulators before infection with HEVcc (p6) for 8 h under modulator treatment, following a medium change and 5 d infection in medium without virus inoculum or treatment. A total of 50 µM of Rbv was renewed after the medium change 8 h postinfection (p.i.), hereby serving as control of efficient inhibition. (B) Quantification of HEVcc (p6) attachment under EGFR modulator treatment in FFU/well in HepG2/C3A cells. Cells were pretreated with EGFR modulators for 30 min at 37 °C before the addition of the virus for 2 h on ice, allowing attachment but not entry. HEV inhibitor Rbv served as negative control here and anti-HEV serum (1:200) as positive control neutralizing HEVcc (p6). Cells were washed thrice before incubation without additives for 5 d p.i. (C) Quantification of effects of EGFR modulators on HEVcc postattachment steps in ORF2 protein positive cells per well in HepG2/C3A cells. Cells were incubated on ice for 30 min before infection with HEVcc (p6) on ice for 2 h. Inoculum was removed, and modulator was added to the cells for the indicated time (8 h or 3 d), allowing modulation only during postattachment processes. (D) HEV p6 replication levels in RNA subgenomic replicon (SGR) system at 72 h post electroporation in HepG2 cells under modulator treatment. (E) Viral titers of HEVcc (p6) produced after electroporation of HEV Kernow-p6 RNA into HepG2 cells after virus production of HEVcc (p6) under modulator treatment, thereby excluding the HEV entry. Titration of progeny virus on HepG2/C3A cells. To test the significance of mean differences, one-way ANOVA, followed by Dunnett multiple comparison test, was used, *p*-values <0.05 (*), <0.01 (**), <0.001 (***), and <0.0001 (****). *p*-values >0.05 were considered to be ns. All assays were performed in triplicates. Mean and SEM are depicted from three independent experiments. Abbreviations: FFU, focus forming units; ns, nonsignificant; ORF2, open reading frame 2; p.i., post infection; RLU, relative light unit; UTC, untreated control cells.

To evaluate whether endogenous EGFR is critical for the entry process of HEV, HepG2 cells were pretreated with the different modulators, followed by infection with HEVcc (p6). The applied drugs and virus inoculum were replaced with fresh medium after 8 hours of incubation. Ribavirin (Rbv) was reapplied to serve as a positive control for the efficient inhibition of HEV replication. By subsequent FFU counting, we identified that Erlo treatment significantly reduced the HEV infection when applied during HEV inoculation (Figure 3A), implying that EGFR is critical for the entry process of HEV.

To dissect whether the restriction of HEV entry by EGFR modulators was based on the restriction of the HEV attachment to the target cells, we incubated modulator-treated HepG2/C3A cells with HEV on ice for 2 hours, allowing virus attachment but not cell entry. Here, anti-HEV serum neutralized HEV particles, thus inhibiting the attachment of HEV. Unbound HEV was removed by repeated washing with PBS, and cells were either directly lysed for RT-qPCR analysis or incubated for 3 days at 37 °C, followed by FFU counting. Thereby, we observed that EGFR modulator treatment did not significantly alter HEV RNA copy numbers (Supplemental Figure S5A, http://links.lww.com/HEP/C666) nor the number of FFU per well (Figure 3B), suggesting that the modulation of endogenous EGFR does not influence the attachment of HEV particles.

To address the role of EGFR on the postbinding steps of HEV, we conducted a postattachment assay by inoculating precooled HepG2/C3A cells with HEV on ice for 2 hours. Inoculum was removed, and cells were treated with EGFR modulators for either 8 hours postinfection (p.i.) or 3 days p.i. at 37 °C. HEV infection was quantified at 3 days p.i., showing a significant reduction in HEV FFU per well in Erlo-treated cells during HEV postattachment for both 8 hours and 3 days, whereas ribavirin treatment reduced HEV infections only when treated for longer than 8 hours (Figure 3C). These data imply that endogenous EGFR modulation affects postbinding steps of HEV.

To circumvent the HEV entry process and address the possible effects of EGFR modulators on intracellular life cycle steps, we transfected *in vitro*-transcribed (IVT) HEV-*Gaussia luciferase* RNA. Hereby, we utilized the HEV RNA subgenomic replicon system, carrying a luciferase reporter, to monitor HEV RNA replication. We detected that EGFR modulator treatment did not affect HEV RNA replication (Figure 3D). To investigate the potential effects of EGFR inhibitors on HEV progeny virus production, IVT full-length HEV RNA was electroporated into hepatoblastoma cells. By quantification of the progeny virus, we detected similar viral titers in the presence or absence of EGFR modulators (Figure 3E), implying that EGFR modulators do not affect HEV RNA replication and virus assembly.

Overall, our data show that endogenous EGFR affects the HEV entry process and has no effect on viral attachment, replication, and assembly of progeny virus.

### Ectopic EGFR expression facilitates HEV infection

**FIGURE 4:**
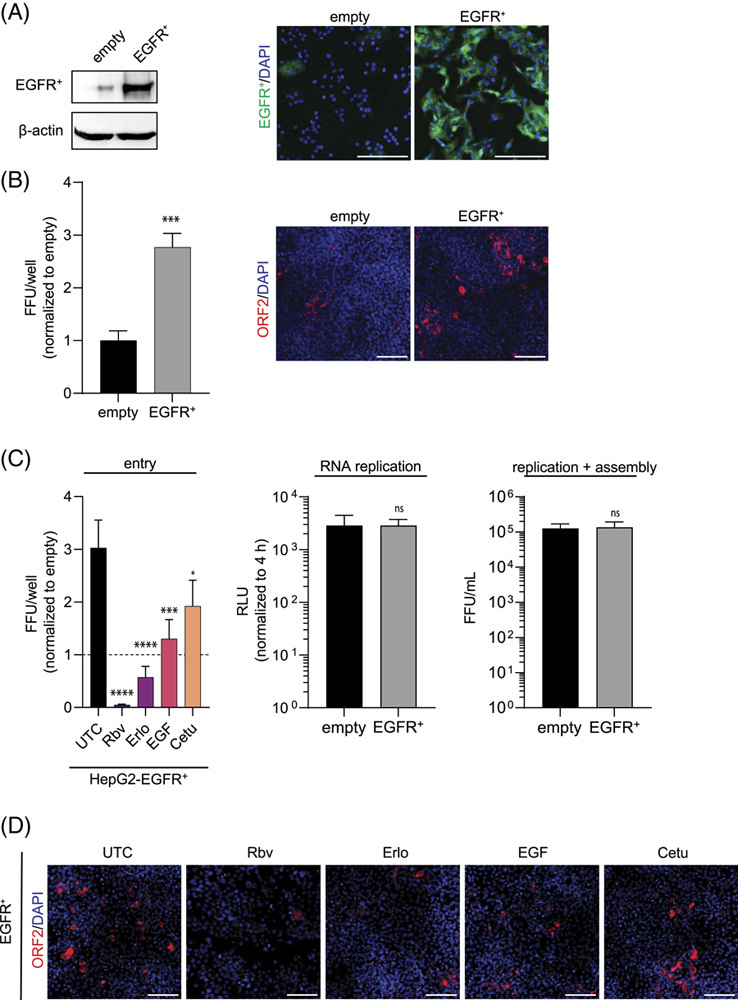
Ectopic EGFR expression facilitates HEV infection and is sensitive to EGFR modulators affecting the HEV entry process. (A) EGFR protein expression in HepG2 cells stably expressing EGFR (HepG2-EGFR) was analyzed by western blot (left) and immunofluorescence (right). Cells transduced with an empty vector served as control. (B) HEVcc (p6) infection in HepG2-EGFR cells. Left: quantification of FFU of the full well normalized to empty vector expressing cells. Right: representative immunofluorescence images stained for ORF2 protein. (C) Right: entry assay of HEVcc (p6) infection in HepG2-EGFR under EGFR modulator treatment [erlotinib (33 µM, Erlo), EGF (16.5 nM), and cetuximab (34 nM, Cetu)] compared with UTC, whereas the HEV inhibitor ribavirin (50 µM, Rbv) served as the control for efficient inhibition. Cells were pretreatment for 30 min with EGFR modulators before infection with HEVcc (p6) for 8 h under modulator treatment, following a medium change and 5 d infection in medium without virus inoculum or treatment. Rbv (50 µM) was replenished, as it served as the control for efficient inhibition. The dashed line indicates the level of normalized FFUs/well of untreated empty vector cells. Middle: HEV (p6) replication level in RNA subgenomic replicon (SGR) system 72 h p.e. in HepG2-EGFR cells normalized to RLU levels at 4 h p.e. Right: HepG2-EGFR cells transfected with HEV Kernow-p6 RNA for virus production. Virus titers determined from non-enveloped virus produced in HepG2-EGFR cells. (D) Representative immunofluorescence images stained for ORF2 protein in HEVcc (p6) infected HepG2-EGFR cells under EGFR modulator treatment during entry, corresponding to the left panel of (C). To test the significance of mean differences, Student *t* test (B and C middle and right) and one-way ANOVA, followed by Dunnett multiple comparison test (C left panel), was used. *p*-values <0.05 (*), <0.01 (**), <0.001 (***), and <0.0001 (****). *p*-values >0.05 were considered to be ns. All infection experiments were performed in triplicates. Mean and SEM are depicted from at least 3 independent experiments. Scale bars = 100 µm. Abbreviations: FFU, focus forming units; ns, nonsignificant; ORF2, open reading frame 2; RLU, relative light unit; UTC, untreated control cells.

To further evaluate the role of EGFR during HEV entry, we generated HepG2 cells ectopically expressing EGFR. After confirming the ectopic expression of EGFR by western blot and immunofluorescence staining (Figure 4A), we challenged the cells with HEVcc (p6). Thereby, we observed an increase in HEV infection in the presence of EGFR compared with HepG2 empty vector cells (Figure 4B). By utilizing the HEV RNA subgenomic replicon and by producing HEVcc in these cells, we further confirmed that ectopically expressed EGFR rather facilitates the initiation of HEV infection without affecting HEV RNA replication and progeny virus production (Figure 4C middle and right).

Given that the modulation of endogenous EGFR restricted HEV entry, we next asked whether the proviral effect of ectopically expressed EGFR is sensitive to EGFR modulator treatment. Importantly, we identified that the proviral effect of EGFR overexpression could be reversed by applying the EGFR-specific modulators during HEV inoculation (Figure 4C left and Figure 4D). Similar to the inhibition of endogenous EGFR, we observed that especially Erlo restricted HEV infection in these assays. Furthermore, we performed an attachment assay, similar to Figure 3B, with cells ectopically expressing EGFR and control cells to quantify HEV RNA copy numbers after viral inoculation at 4 °C for 2 hours. We detected similar HEV RNA copy numbers in EGFR-expressing cells and control cells, indicating that EGFR does not affect HEV attachment (Supplemental Figure S5B, http://links.lww.com/HEP/C666). Taken together, our obtained data suggest that access to EGFR is a limiting parameter for the initiation of HEV infection.

### EGFR facilitates HEV infection independent of its kinase activity

**FIGURE 5:**
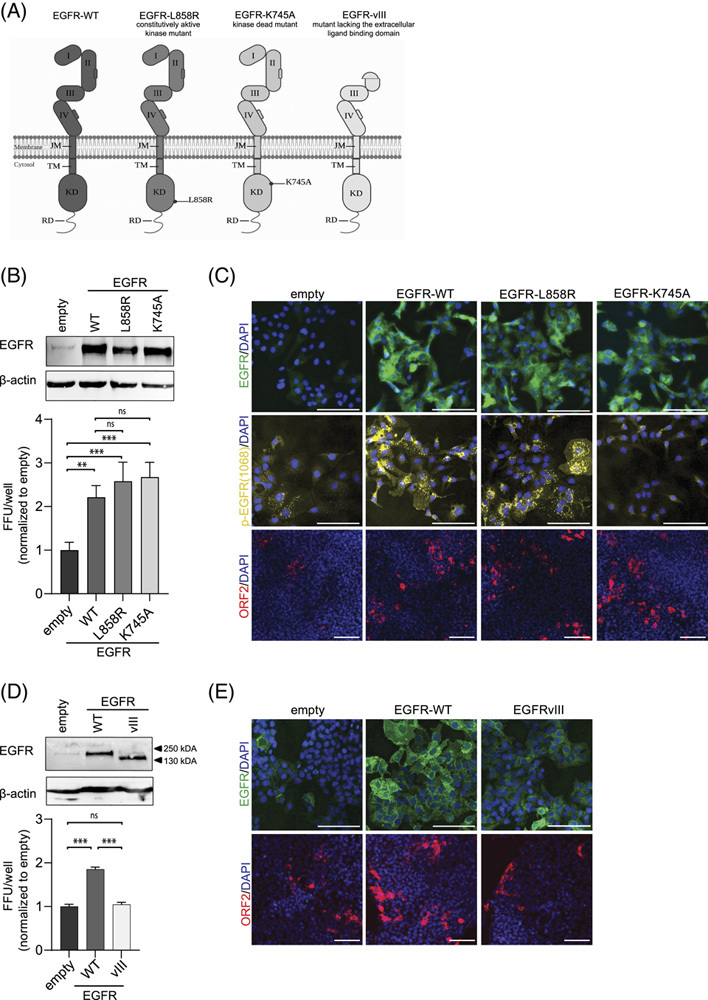
Ectopic expression of EGFR mutants indicates no effect of EGFR signaling in HEV infection. (A) Schematic diagram of EGFR (-mutant) domains. (B) EGFR protein expression in HepG2 cells ectopically expressing EGFR (-mutants) was analyzed by western blot (upper). HEVcc (p6) infection in EGFR (-mutant) ectopically expressing HepG2 cells (lower). Quantification of FFUs/well normalized to cells stably expressing only the empty vector. (C) Immunofluorescence analysis of EGFR protein expression (upper) and EGFR phosphorylation at Tyr1068 after overnight FCS starvation of EGFR (-mutant) ectopically expressing HepG2 cells and challenge with EGF (16.5 nM) for 15 min (middle). Representative fluorescence images of HEVcc (p6) infection in EGFR (-mutant) expressing cells after staining against ORF2 protein (lower). (D) EGFR protein expression in HepG2 cells ectopically expressing the EGFR-WT or EGFRvIII mutant analyzed by western blot (upper). HEVcc (p6) infection in EGFR (-mutant) ectopically expressing HepG2 cells (lower). Quantification of FFUs/well normalized to cells stably expressing only the empty vector. (E) Immunofluorescence analysis of EGFR protein expression (upper) and representative fluorescence images of HEVcc (p6) infection in EGFR (-mutant) expressing cells after staining against ORF2 protein (lower). Infection experiments were performed in triplicates. To test the significance of mean differences, one-way ANOVA, followed by Dunnett multiple comparison test, was used. *p*-values <0.05 (*), <0.01 (**), <0.001 (***), and <0.0001 (****). *p*-values >0.05 were considered to be ns. Mean and SEM are depicted from 3 independent experiments. Scale bars = 100 µm. Abbreviations: EGFR, EGF receptor; FFU, focus forming units; JM, juxtamembrane domain; KD, kinase domain; ns, nonsignificant; RD, regulatory domain; TM, transmembrane domain; WT, wild type.

To gain additional insight into whether EGFR kinase activity and signaling are relevant for HEV infection and entry, we stably expressed the EGFR mutants EGFR-L858R and EGFR-K745A in HepG2 cells. While a mutation of leucine at position 858 to arginine leads to constitutive activation of EGFR kinase, the mutation of lysine 745 to alanine impairs its kinase function (Figure 5A).^33^,^34^ After confirmation of the ectopic expression of EGFR and its mutants by western blot and immunofluorescence staining (Figure 5B, C upper panels), the activity levels of the EGFR kinase domains were evaluated (Figure 5C middle panel). Therefore, the respective cells were serum starved overnight, followed by the addition of EGF (16.5 nM or 100  ng/mL for 15 minutes. An immunofluorescence staining was performed to determine the phosphorylation status at Tyr1068. Although only a few pEGFR(1068) signals were observed in the cytoplasm of empty vector cells and cells ectopically expressing the EGFR-K745A mutant, a high abundance of pEGFR(1068) signals was observed in EGFR and EGFR-L858R expressing cells (Figure 5C middle panel). To demonstrate the ability of the EGFR-L858R mutant to signal even in the absence of ligand binding, we performed the immunofluorescence analysis of EGFR’s phosphorylation status at Tyr1068 after overnight serum starvation. A high abundance of pEGFR(-1068) signals was visible even without ligand induction in EGFR-L858R HepG2 cells, whereas only very low levels of pEGFR(-1068) signals were visible in EGFR-WT cells (Supplemental Figure S6, http://links.lww.com/HEP/C666). Hereby, we validated the constitutively active kinase function of EGFR in EGFR-L858R HepG2 cells and its inactivation in EGFR-K745A HepG2 cells.

We next tested whether the different EGFR mutants were capable to facilitate HEV infection compared with EGFR wild type (EGFR-WT). To this end, the respective cell lines were infected with HEV, and the HEV infection was quantified through immunofluorescence staining. Similar to the EGFR-WT, we detected significantly increased HEV infection in the presence of both EGFR-L858R and EGFR-K745A, compared with empty vector control (Figure 5B, C lower panels), implying that EGFR facilitates HEV infection independent of its kinase activity. Furthermore, we observed no effect on HEV RNA replication nor on HEV progeny virus production after the electroporation of HEV IVT RNA in the presence of the different EGFR mutants (Supplemental Figure S7A–B, http://links.lww.com/HEP/C666). In summary, our results suggest that access to EGFR is critical for HEV and facilitates infection independent of its kinase activity.

To analyze whether the extracellular EGFR interaction plays a role in HEV infection, we utilized an EGFR mutant lacking its ligand-binding domain (EGFRvIII) while obtaining constitutively low levels of active signaling.^35^ We first validated the ectopic expression of EGFR-WT and EGFRvIII mutant in HepG2 cells by western blot and immunofluorescence (Figure 5D, E upper panels).

Subsequently, we tested whether the EGFRvIII mutant was capable to facilitate HEV infection by infecting the respective cells with HEVcc (p6) and quantification of HEV infection via immunofluorescence. Of note, we observed a significant increase of HEV infection in the presence of EGFR-WT but not in the presence of EGFRvIII compared with empty vector cells, suggesting that the lack of the EGF-binding domain abolishes the EGFR-mediated proviral effect. Furthermore, we detected no effect on HEV RNA replication nor on HEV progeny virus production after the electroporation of HEV IVT RNA in the presence of the different EGFR mutants (Supplemental Figure S7C–D, http://links.lww.com/HEP/C666). Taken together, the obtained data imply that the EGFR ligand-binding domain plays a crucial role in facilitating HEV infection.

### EGFR is critical for HEV entry in HepaRG cells and PHHs

**FIGURE 6:**
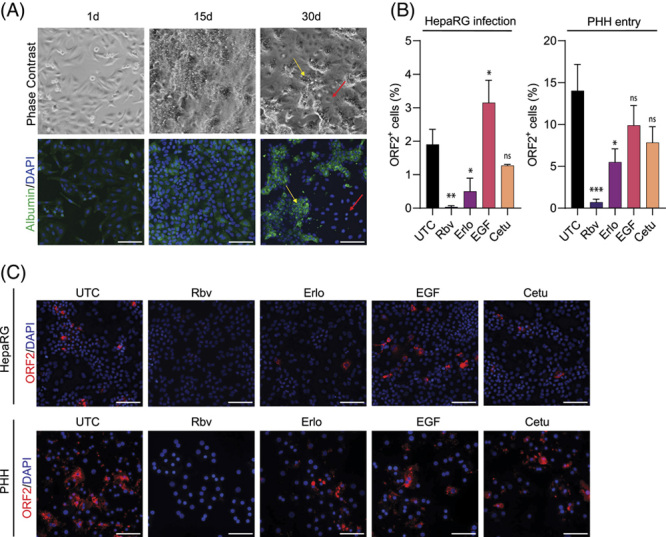
The critical entry effect of EGFR was verified in HepaRG cells and PHHs. (A) Phase contrast and immunofluorescence images of HepaRG cells during differentiation. The yellow arrow indicates hepatocyte-like cells, red arrow cholangiocyte-like cells. (B, C) HEVcc (p6) infection in differentiated HepaRG cells under EGFR modulator treatment during the full infection time of 5 d or PHH only during entry, meaning a 30 min pretreatment with EGFR modulators before infection with HEVcc (p6) for 16 h under modulator treatment, after 3 d incubation time without inoculum or modulators. [erlotinib (33 µM, Erlo), EGF (16.5 nM), and cetuximab (34 nM, Cetu) compared with UTC, whereas 25 µM of the HEV inhibitor ribavirin (Rbv) served as control]. (B) Quantification of ORF2-positive cells by CellProfiler analysis in percent of all counted DAPI nuclei per image and (C) representative fluorescence images stained against ORF2 protein. Infection experiments were performed in duplicates, with at least 10 images taken for analysis per experiment. To test the significance of mean differences, one-way ANOVA, followed by Dunnett multiple comparison test (D), was used, *p*-values <0.05 (*), <0.01 (**), <0.001 (***), and <0.0001 (****). *p*-values >0.05 were considered to be ns. Mean and SEM are depicted from 2 (HepaRG) or 4 (PHH) independent experiments. Scale bars = 100 µm. Abbreviations: ns, nonsignificant; ORF2, open reading frame 2; PHH, primary human hepatocyte; UTC, untreated control cells.

To further validate our findings in a cell culture model exhibiting more characteristics of hepatocytes *in vivo*, differentiated HepaRG cells were used. HepaRG cells exhibit many key metabolic enzymes and receptors that make them an attractive alternative model for *in vitro* studies and, as such, have already been extensively used in the study of other hepatotropic viruses.^36^,^37^ Therefore, HepaRG cells were differentiated into cholangiocyte-like and hepatocyte-like cells.^29^ Successful differentiation was confirmed by immunofluorescence staining for the hepatocyte marker albumin (Figure 6A). To address the importance of EGFR in this cell culture system, we infected differentiated HepaRG cells with HEVcc (p6) in the presence of EGFR modulators and subsequently determined ORF2+ cells (Figure 6B left and Figure 6C upper panel). We detected that Erlo efficiently reduced HEV infection. In contrast to our previous findings in hepatoblastoma cells, EGF increased HEV infection.

To evaluate whether EGFR modulators are capable of restricting HEV entry in primary cells, we pretreated PHHs with EGFR modulators, followed by HEVcc (p6) infection. Both Erlo and Cetu reduced HEV infection by ~61% and 55%, respectively, when compared with untreated cells (Figure 6B right and Figure 6C lower panel), highlighting the potential restriction capacity of EGFR modulators during HEV infection *ex vivo*. Taken together, these data suggest that EGFR is critical for HEV entry in primary cells and that HEV infection can be restricted by the application of EGFR modulators during HEV inoculation.

## DISCUSSION

Although HEV is an increasing health burden, knowledge of HEV’s pathogenesis and life cycle has been scarce so far. Despite the fact that HEV entry is an appealing target for pharmacological intervention, druggable host factors to prevent HEV entry have yet to be identified.^12^,^13^ EGFR is a receptor tyrosine kinase and, as such, involved in cell migration, proliferation, and differentiation.^20^,^21^ Importantly, EGFR has been found to be a host factor for numerous viruses affecting different life cycle steps. Viruses like severe acute respiratory syndrome coronavirus 2 (SARS-CoV-2)^38^ and Epstein-Barr virus^39^ among others regulate EGFR expression and recycling, thereby isolating host cells from host-specific signals forcing them to respond solely to viral signals and thus optimizing cellular environments for productive infections. Other viruses, including influenza A virus,^40^ rhinoviruses, and respiratory syncytial virus,^41^ manipulate EGFR signaling to antagonize viral inflammation and host antiviral systems. Furthermore, EGFR signaling is utilized for viral entry and replication by remodeling the actin network enabling entry (ie, human cytomegalovirus,^42^ herpes simplex virus 1^43^) or inducing favorable environments for replication (ie, Epstein-Barr virus^44^). In addition, EGFR’s trafficking is exploited by HCV^18^ and HBV,^45^ thereby facilitating cell entry by linking the virus-host cell complex to the endocytic machinery. Finally, EGFR can act as a coreceptor stabilizing virus-host cell complexes or enriching initial or sequential receptors. In this study, we present EGFR as a new host factor for HEV in human hepatocytes.

First of all, we found that endogenous EGFR is abundantly expressed in hepatocytes and cholangiocytes in the human liver *in vivo* (Figure 1) and plays a role in HEV infections using EGFR-specific siRNA and the EGFR kinase inhibitor Erlo (Figure 2). We were able to confirm EGFR’s role in iPSC-derived HLCs through shRNA knockdown. Furthermore, different strains of HEV (p6 and 83-2), as well as nonenveloped and enveloped HEV, were significantly affected by EGFR inhibition, albeit to slightly different degrees. Our findings that ectopic expression of EGFR (Figure 4) increases HEV infections further implies that EGFR is critical for HEV. To dissect the effect of EGFR in the HEV life cycle, we performed assays specific for each step (Figure 3). Here, we were able to show that the effect is specific to the entry process while leaving the attachment, replication, and assembly unaltered. We further analyzed the impact of EGFR kinase activity on HEV infectivity using a constitutively active kinase mutant and a kinase-dead mutant. The expression of the two EGFR mutants, the constitutively active and the kinase-dead mutant, both facilitate HEV infections, which implies that the kinase function does not affect the HEV life cycle. However, by deprivation of EGFR’s ligand-binding domain (EGFRvIII mutant), the proviral effect of EGFR is lost, underlining the crucial role of its ligand-binding domain in HEV infection. We, therefore, suggest that the receptor itself or noncanonical pathways modulate HEV entry. At this stage of understanding, two possible mechanisms are likely for EGFR’s effect on HEV entry: (1) by utilizing EGFR’s trafficking or (2) by EGFR as a coreceptor. For example, EGFR endocytosis and trafficking are hijacked by HBV.^45^ There, the EGFR endocytic machinery drives the translocation of HBV-receptor (NTCP)-bound HBV from the cell surface through the endosomal network to late endosomes and lysosomes, thus providing an entry mechanism. However, this mechanism is rather unlikely to be the reason for EGFR’s effect on HEV entry as the EGFR modulators and EGFR mutants modulate EGFR trafficking in a different manner but show similar effects,^46^–^49^ and HEV is found to be internalized depending on clathrin-mediated endocytosis.^15^ For example, although Erlo and Cetu have been found to induce caveolin-mediated internalization of EGFR, low concentrations (<2 ng/mL) of EGF activate EGFR endocytosis in a clathrin-dependent manner. Even in the presence of higher concentrations (100 ng/mL, 16.5 nM) of EGF, roughly 60% of the receptor have been reported to be endocytosed clathrin-mediated.^46^–^49^ Given the differences in the internalization routes of different EGFR variants and the crucial role of EGFR’s extracellular ligand-binding domain, as well as the proposed distinct entry mechanisms for enveloped and non-enveloped HEV,^50^ we speculate a mechanism that involves EGFR as an entry cofactor. EGFR could either increase the binding avidity to an initial yet unknown receptor or sequential proteins important for the entry of enveloped and nonenveloped HEV. Alternatively, EGFR could associate to or stabilize an initial receptor or sequential proteins. Furthermore, the enrichment of initial receptors important for HEV’s entry could be a possible mechanism of EGFR as a cofactor as well. EGFR modulators might lower the surface expression of EGFR or disrupt associations of EGFR with entry receptor(s) of HEV, thus decreasing HEV infection.^46^ For instance, the EGFR kinase inhibitor Erlo impacts EGFR’s localization and other nonsignaling pathways in addition to inhibiting its classical signaling, which causes EGFR to be arrested inside the cell and degraded.^46^ Furthermore, EGF can induce EGFR activation and internalization, reducing the surface level of EGFR temporarily.^24^,^25^ However, proving the proposed mechanisms is not possible yet, as the main receptor needed for HEV entry has not been identified so far and knowledge on its entry process is scarce,^12^,^13^ thus limiting available assays.

In conclusion, our study revealed EGFR as a novel host factor for HEV’s entry process in hepatoblastoma-cell culture systems and also *ex vivo* in PHHs, underlining the relevance of this particular factor. EGFR’s kinase function and canonical signaling were found to be of no influence, whereas EGFR’s ligand-binding domain was found to be crucial for the facilitation of HEV infection. Therefore, EGFR is likely an entry cofactor, either increasing the binding avidity, stabilizing, or associating with initial HEV receptors (or sequential proteins), thus bringing the initial receptor (or sequential proteins) in close proximity. Alternatively, EGFR could augment necessary receptors and thus facilitating viral entry. However, future studies have to address the identification of the initial receptor(s) to implement novel assays studying the receptor’s specific role. Importantly, EGFR has been identified to play a diverse range of roles in viral infection; its participation in HEV infections therefore broadens its scope and gives not only great advances in the understanding of EGFR’s role in viral infections but also substantially expands the scarce knowledge of HEV host factors.

## Supplementary Material
