# Supplementary material for: EGF receptor modulates HEV entry in human hepatocytes
Source: Hepatology. 2023 Feb 7;77(6):2104–17. doi: 10.1097/HEP.0000000000000308 (PMC10187617; doi:10.1097/HEP.0000000000000308)
Supplement: Supplementary file 1 [file hep-77-2104-s001.pdf]

Supplementary Information – HEP-22-1113

Epidermal Growth Factor Receptor Modulates Hepatitis E Virus Entry in Human Hepatocytes

Jil Alexandra Schrader[1], Thomas Leon Burkard[1], Yannick Brüggemann[1], André Gömer[1], Toni Luise Meister[1], Rebecca Menhua Fu[2,3], Ann-Kathrin Mehnert[2,3], Viet Loan Dao Thi[2,4], Patrick Behrendt[5,6,7], David Durantel[8], Ruth Broering[9], Florian W. R. Vondran[10], Daniel Todt[1,11], Volker Kinast[1,12#], Eike Steinmann[1,113#]

[1] Ruhr University Bochum, Institute for Hygiene and Microbiology, Department for Molecular and Medical Virology, Bochum, Germany

[2] Schaller Research Group at Department of Infectious Diseases and Virology, Heidelberg University Hospital, Cluster of Excellence CellNetworks, Heidelberg, Germany

[3] Heidelberg Biosciences International Graduate School, Heidelberg University, Heidelberg, Germany

[4] German Centre for Infection Research (DZIF), Partner Site Heidelberg, Heidelberg, Germany

[5] TWINCORE Centre for Experimental and Clinical Infection Research, a Joint Venture between the Medical School Hannover (MHH) and the Helmholtz Centre for Infection Research (HZI), Institute for Experimental Virology, Hannover, Germany

[6] Hannover Medical School, Department of Gastroenterology, Hepatology and Endocrinology, Hannover, Germany

[7] German Centre for Infection Research (DZIF), Partner Site Hannover - Braunschweig, Hannover, Germany

[8] CIRI - Centre International de Recherche en Infectiologie, Univ Lyon, Université Claude Bernard Lyon 1, Inserm, U1111, CNRS, UMR5308, ENS Lyon, Lyon, 69007, France.

[9] University Hospital Essen, University Duisburg-Essen, Department of Gastroenterology, Hepatology and Transplant Medicine, Essen, Germany

[10] Hannover Medical School, Department of General, Visceral and Transplant Surgery, Hannover, Germany;

[11] European Virus Bioinformatics Centre (EVBC), Jena, Germany

[12] Carl von Ossietzky University Oldenburg, Department of Medical Microbiology and Virology, Oldenburg, Germany

[13] German Centre for Infection Research (DZIF), External Partner Site, Bochum, Germany

Material and Methods

Hepatocyte-Like Cells (HLCs)

Hepatocyte-like cells were made from induced pluripotent stem cells (iPSCs) as described previously[1] and plated for differentiation on a 48-well plate.

Production of Ectopically Expressing Cell Lines via Lentiviral Transduction

For the production of lentiviral particles, 8×10^5 293T cells were seeded on collagen-coated 6-well plates. The following day, the 293T cells were transfected with the plasmids pcz-VSV-G, pCMV-dR8.74 and pWPI-BLR with the respective gene of interest using Lipofectamine 2000 (Invitrogen, Cat. Nr. 11668019) following the manufacturer's instructions. Six hours post transfection, a medium change was done and the lentiviral particles were harvested 48 h and 72 h post transfection by collecting the supernatant and filtering it through a 0.45 µm mesh (Filtropur 0.45, Sarstedt, Cat. Nr. 83.1826) to remove any cell debris. 1×10^5 target cells (HepG2) per well were seeded on a 6-well plate prior to treatment with 4 µg/mL polybrene (Sigma-Aldrich, Cat. Nr. TR-1003) and infection with 1 mL of lentiviral particles for 6–8 h. Selection of the transduced cells was started 48 h post transduction using 5 µg/mL blasticidin-S-hydrochlorid (Fisher Bioreagents, Cat. Nr. BP2647) and further maintained in DMEM complete containing blasticidin-S-hydrochlorid. Validation of the ectopic expression was done via western blot and immunofluorescence staining.

siRNA-Mediated Knock Down

Knock down of endogenously expressed EGFR was performed using two validated short interfering RNAs (siRNA, Ambion, ID s564 and s565, Cat. Nr. 4427038) and a control siRNA (Ambion, Silencer Select, Cat. Nr. 4390843). 0.5 µM each of the siRNA were transfected using Lipofectamine RNAiMax (Invitrogen, Cat. Nr. 13778) following the manufacturer's instructions. Two days post transfection, the cells were either subjected to infection assays or lysed for western blot analysis.

shRNA-Induced Knock Down via Adeno-Associated Viral (AAV) Transduction

The sequence of the shRNA targeting EGFR was obtained from Rothenberg *et al.*[2](sequence of shEGFR: GCTGGATGATAGACGCAGATA, sequence of shCtrl: GGTCGTGAACTAATCAGAGGA). The forward strand oligonucleotides were designed by starting with a 5´CACC overhang for cloning using BsmbI, followed by the sense sequence, a connecting 7 nt loop (TCAAGAG) sequence and finally the antisense sequence (targeting sequence). The reverse strand was designed using a 5´AAAA overhang. The shRNA genes were cloned into a self-complementary AAV6 vector under the U6 promoter. Iodixanol purified and recombinant AAVs were produced as described previously[3]. HLCs were transduced with AAVs three days prior to HEV infection. The inoculum was removed 24 h post transduction. The cells washed once with PBS and fresh HLC culture medium renewed.

In Vitro Transcription and Electroporation

Before *in vitro* transcription, HEV Kernow-C1-p6-full length and -Gluc plasmid were linearized using MluI (New England Biolabs, Cat. Nr. R3198), while HEV 83-2-containing plasmids were linearized with HindIII (New England Biolabs, Cat. Nr. R3104). *In vitro* transcribed RNA (IVTs) were produced as described in Todt et al. [4]. IVTs were subsequently transfected into the respective cells using the electroporation technique described in [4]. Shortly, 5 µg of *in vitro* transcribed RNA were mixed with 5×10^6 cells in 400 µL cytomix containing 2 mM ATP (Cayman Chemical, Cat. Nr. 14498) and 5 mM glutathione (Sigma Aldrich, Cat. Nr. # G4251). After electroporation using the Gene Pulser system (Bio-Rad), cells were immediately transferred to 12.1 mL of DMEM complete and the cell suspension was seeded in respective plates depending on the experiment (2×10^4 cells/well seeded in a 96-well plate for luciferase assays, 12.5 mL seeded in a 10 cm dish for virus production).

Production of Cell Culture-Derived HEV Particles (HEV_{CC})

HEVcc (p6 and 83-2) was produced as previously described [4]. In brief, HEV IVTs were electroporated into HepG2 cells. Seven days post electroporation, the supernatant containing enveloped HEVcc was filtered through a 0.45 µm mesh (Filtropur 0.45, Sarstedt, Cat. Nr. 83.1826) and stored at 4 °C for up

to 7 days. To harvest non-enveloped HEVcc, the cells were trypsinized, resuspended in fresh DMEM complete and lysed via three freeze-thaw cycles in liquid nitrogen. The lysate was cleared from cell debris by a 10 000×g centrifugation for 10 min and titrated on HepG2/C3A cells to determine viral titers. Non-enveloped HEVcc was frozen at −80 °C until further usage. Cells were fixed with 3% paraformaldehyde (PFA, Roth, Cat. Nr. 93351) seven days post infection for immunofluorescence staining against ORF2 protein and determination of the number of focus forming units (FFU) according to Todt et al. [4].

HEV Infection Assays with Non-Enveloped HEVcc

For infection assays, either 3.5×10^3 HepG2(-derived) cells were seeded on collagen-coated 96-well plates, or differentiated HepaRG cells (24-well plate) or PHHs seeded at 5×10^5 cells/well were used (see section *cell culture*). The following day, cells were infected with a multiplicity of infection (MOI) of 0.5–2 or 1×10^5 FFU per well (HLC). Erlotinib (33 µM, MedChemExpress, Cat. Nr. HY-50896), EGF (16.5 nM, 100 ng/mL, MedChemExpress, Cat. Nr. HY-P7109), Cetuximab (34 nM, MedChemExpress, Cat. Nr. HY-P9905) and Ribavirin (50 µM, Sigma-Aldrich, Cat. Nr. R9644) were applied simultaneously to virus inoculum. A medium change of infected PHHs was performed 16 h post infection (p.i.) to fresh William's medium E with supplements for PHHs, Rbv was reapplied. The medium was changed of infected HLCs at 24 h p.i.. PHHs were fixed 3 d p.i.with 3% PFA and HLCs 7 d p.i.while other assays were fixed 5 d p.i. for immunofluorescence staining of ORF2 protein and determination of FFUs/well or % ORF2 protein positive cells/image section.

HEV Infection Assays with Enveloped HEVcc

For infection assays using enveloped HEVcc, either 1×10^4 HepG2/C3A cells were seeded on collagen-coated 96-well plates one day prior to infection with 200 µL/well of enveloped HEVcc (MOI 0.02–0.05). The inoculum was removed 24 h p.i. and fresh MEM complete supplied. The indicated modulators were applied in the inoculum at the time of infection as well as renewed and applied into the fresh MEM complete after the medium change. Cells were fixed 5 d p.i. or immunofluorescence staining of ORF2 protein and determination of FFUs/well.

HEV Attachment Assay

For attachment assays, 1×10^4 HepG2/C3A cells were seeded on collagen-coated 96-well plates. The following day, the cells were pretreated with EGFR modulators for 30 min at 37 °C and the plate then put on ice for 30 min before addition of ice-cold non-enveloped HEVcc(p6) (MOI 1–2) and the tested modulator and incubation for another 2 h on ice. Afterwards, the medium was removed and the cells washed thrice with ice-cold PBS before either the RNA was isolated for qPCR analysis using the RNasy Mini Kit (Qiagen, Cat. Nr. 74104) following manufacturer's instructions or the cells were supplied with fresh MEM complete and incubated for 5 days at 37 °C in a 5% (v/v) CO_2 incubator. The cells were then fixed with 3% PFA and subjected to an immunofluorescence staining of ORF2 protein and determination of FFUs/well as described below.

HEV Postbinding Assay

For postbinding assays, 1×10^4 HepG2/C3A cells were seeded on collagen-coated 96-well plates. The following day, the plate was incubated on ice for 30 min prior to infection with non-enveloped HEVcc p6 (MOI 0.5–2) on ice. The inoculum was left on the cells on ice for 2 h before removal. The cells were washed thrice with ice-cold PBS and fresh medium supplied with the respective modulator was added and the cells incubated at 37 °C in a 5% (v/v) CO_2 incubator for either 8 h or 3 days. The medium was changed at the indicated time and the cells washed with PBS thrice and fresh medium without modulators was added. The cells were then incubated at 37 °C in a 5% (v/v) CO_2 incubator until fixation 3 d p.i. with 3% PFA and subjected to an immunofluorescence staining of ORF2 protein and determination of FFUs/well as described below.

HEV Entry Assay

For entry assays, 3.5×10^3 HepG2(-derived) cells were seeded on collagen-coated 96-well plates. The following day, cells were pretreated for 30 min with the respective modulator before infection with non-enveloped HEVcc (MOI 0.5–2) for 6–8 h. Medium was removed and fresh DMEM complete with neither virus nor modulator was added except for ribavirin (Rbv), which was added into the fresh

medium again. Cells were incubated for 5 d p.i. before fixation with 3% PFA, immunofluorescence staining of ORF2 protein and determination of FFUs/well as described below.

HEV Luciferase Replication Assays

HEV replication was monitored using a *Gaussia* luciferase (Gluc) construct replacing the ORF2 in the HEV Kernow-C1-p6 genome [5]. In order to measure the luciferase activity, the respective cells were electroporated with HEV Kernow-p6-Gluc IVTs. 20 µL of the supernatant were collected at the indicated hours post electroporation (h p.e.) and transferred to a white, flat-bottom microplate (Greiner Bio-One, Cat. Nr. 655074). The supernatant was subsequently incubated with luciferase substrate (1 µmol/L of coelenterazin in PBS, Carl Roth, Cat. Nr. 4094.3) and luciferase activity was measured in a luminometer (CentroXS3 LB960, Berthold technologies).

RT-qPCR

HEV RNA was quantified using a TaqMan® probe (5'-6FAM-TGATTCTCAGCCCTTCGC-BBQ-3') one step RT-qPCR based on the GoTaq® Probe 1-Step RT-qPCR System (Promega). 50 ng of isolated RNA were used as template and 5'-GGTGGTTTCTGGGGTGAC-3' (sense) and 5'-AGGGGTTGGTTGGATGAA-3' (antisense) as HEV primers were utilized[6]. An RNA transcript served as a standard to quantify RNA copy numbers by serial dilution[4]. All RT-qPCR were run on a LightCycler 480 system (Roche).

Immunofluorescence Staining and Microscopy

Cells were fixed by applying 3% PFA for at least 10 min followed by permeabilization in 0.1% Triton X-100 (Carl Roth, Cat. Nr. 3051.3) in 1× PBS for 5 min. Subsequently, the cells were blocked in 5% horse serum (Gibco, Cat. Nr. 26050-088) in 1× PBS for a minimum of 1 h. EGFR expression was stained with a polyclonal goat antibody (R and D Systems, Cat. Nr. AF231. 1:500 in 5% horse-serum), EGFR phosphorylation at Tyr1068 was visualized with monoclonal rabbit antibody (Cell Signaling Technology, Cat. Nr. 3777S, 1:200 in 5 % horse-serum), for staining of albumin polyclonal anti-rabbit antibody (Agilent, Cat. Nr. A0001, 1:500 in 5% horse-serum) was used, and for the capsid protein

(ORF2 protein) a polyclonal HEV genotype 3 capsid protein-specific rabbit hyperimmune serum (diluted 1:5 000 in 5% horse serum, kindly gifted by Prof. Rainer G. Ulrich, Friedrich Loeffler Institute, Germany [7]) was used and cells with the respective antibody incubated at 4 °C on a rocking shaker overnight. Unbound primary antibody was removed by washing twice with 1× PBS and the secondary antibody (goat anti-rabbit AlexaFluor 488 or rabbit-anti-goat AlexaFluor 488, 1:1 000 in 5% horse serum, Invitrogen, Cat Nr. A-11008 and A-11078, respectively) was added. After 2 h in the dark on a rocking shaker, the cells were washed twice with 1× PBS and the DNA labelled with either 4′,6′-diamidino-2-phenylindole (Invitrogen, Cat. Nr. D1306, DAPI, 1:10,000 in H_2O) for 5 min or Hoechst 33342 (Thermo Fisher Scientific, 1:1000 in H_2O). Afterwards, the cells were washed twice with water and stored in water at 4 °C until imaging. All staining steps were performed at room temperature unless otherwise stated. Images of fluorescently stained cells were taken with a Keyence BZX800 microscope with 4×, 10× or 20× objectives or with the Zeiss Cell discoverer 7 (CD7) microscope.

Western Blots

For western blot analysis, cells were lysed in either M-Per buffer (Thermo Scientific, Cat. Nr. 78501) supplemented with Pierce Protease Inhibitor Mix (Thermo Scientific, Cat. Nr. A32953) or RIPA buffer containing the cOmplete Mini Protease Inhibitor Cocktail (Roche). Cells were centrifuged at 10 000×g for 15 min, the supernatant then heated at 95°C for 5 min with 1× Laemmli-Buffer and resolved by SDS-PAGE before being transferred to either nitrocellulose or polyvinylidene fluoride (PVDF) membranes by wet tank electroblotting. Membranes were blocked with 5% milk in PBS containing 0.05% Tween (PBS-T) for min. 1 h at room temperature and subsequently incubated with primary rabbit monoclonal anti-EGFR antibody (Cell Signaling Technologies, Cat. Nr. 4267S, 1:1000 in 0.5% milk), overnight at 4 °C, followed by incubation with secondary horseradish peroxidase (HRP) conjugated polyclonal goat anti-rabbit antibody (Abcam, Cat. Nr. #ab97051, 1:10 000 or Jackson Immuno Reseach, 1:4 000, in 0.5% milk) for 2 h at room temperature. The primary antibody targeting β-actin (Sigma-Aldrich, Cat. Nr. A3854, 1:10 000 in 0.5% milk,) was already conjugated with HRP and was thus incubated for 2 h after blocking. Subsequently, membranes were developed using the Pierce ECL

Western Blotting Substrate (Thermo Scientific, 32109) and analyzed using a chemiluminescence imaging machine (Celvin S 420, Biostep Sarstedt, or INTASELL Chemostar imager).

Cell Viability Assay

To determine the cell viability upon treatment with the different drugs, an MTT (3-(4,5-dimethylthiazol-2-yl)-2,5-diphenyltetrazolium bromide) assays was performed. For this, 2.5×10^4 HepG2 cells were seeded on collagen-coated 96-well plates. The following day, cells were treated with the indicated amounts of modulator. Three days post treatment, MTT substrate (Biomol, Cat. Nr. 15655) supplied in DMEM complete medium was added to the cells and incubated at 37 °C in a 5% (v/v) CO_2 incubator for 2 hours or until cells have metabolized the substrate to MTT formazan. The medium was removed, 50 µL DMSO added to each well and the absorbance photometrically measured at 570 nm using the Tecan Sunrise Remote plate reader. Cells treated with 70% ethanol served as background control.

scRNA Seq

Single-cell RNA Sequencing data was mined from Aizarani et al. [8]. RNA expression was visualized by T-distributed stochastic neighbor-embedding (t-SNE) plots via the human liver cell atlas webservice (http://human-liver-cell-atlas.ie-freiburg.mpg.de/). Violin plots were computed with in-house R script using the following packages: SingleCellExperiment, Tidyverse, Scater, Scran.

Statistical Analysis and Software

Statistical analysis was performed using GraphPad Prism v9.12 for Windows (La Jolla, CA, USA, www.graphpad.com). Dose-dependent treatment was plotted and adjusted to a non-linear regression mode using GraphPad Prism. To test significance of mean differences, either student t-test or one-way ANOVA followed by Dunnett's multiple comparison test were used, p values < 0.05 (*), <0.01 (**), <0.001 (***) and <0.0001 (****) were considered statistically significant. p values >0.05 were considered to be non-significant (ns). For image analysis Fiji-ImageJ (v1.53q) [9] and CellProfiler (v. 4.0.7) (www.cellprofiler.org) were used. Graphics were prepared using GraphPad Prism v9.12 for

Windows (La Jolla, CA, USA, www.graphpad.com), Adobe Illustrator v26.0.3 (www.adobe.com) and BioRender (www.biorender.com).

Supplementary Tables and Figures

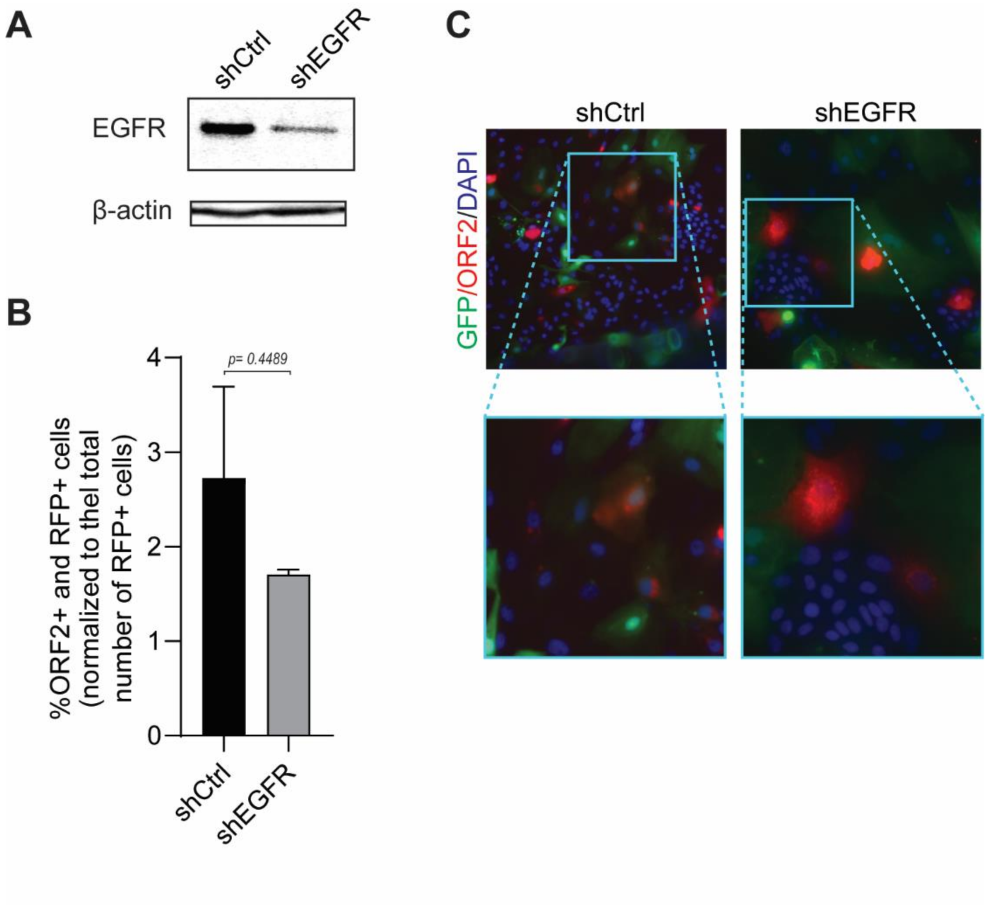

Supplementary Figure S1: Knock down of EGFR in HLCs reduces HEV infection. (A) EGFR protein expression in iPSC-derived HLCs 48 h post transduction with AAVs carrying shRNA targeting EGFR (shEGFR) or control RNA (shCtrl). **(B)** Quantification of HEV-infected and shRNA-transduced (ORF2+, RFP+) HLCs normalized to the total number of HLCs transduced (RFP+). (ORF2+, RFP+) / ((ORF2+, RFP+2) + (ORF2-, RFP+)). **(C)** Representative immunofluorescence images stained for ORF2 protein (red). Transduced cells carrying a GFP reporter are indicated in green. Infection experiments were performed in duplicates with at least 20 frames counted. Mean and SEM are depicted from at least two independent experiments.

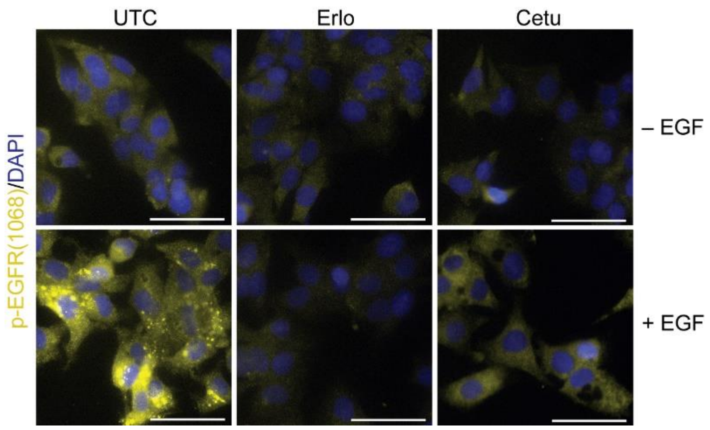

Supplementary Figure S2: Erlotinib and Cetuximab inhibit EGF induced phosphorylation of EGFR at Tyr1068. Immunofluorescence analysis of EGFR phosphorylation at Tyr1068 after overnight FCS starvation of EGFR-WT ectopically expressing HepG2 cells and after treatment with Erlotinib (33 µM, Erlo), or Cetuximab (34 nM, Cetu) for 30 min prior to challenge with EGF (16.5 nM) for 15 min. Scalebars = 100 µm.

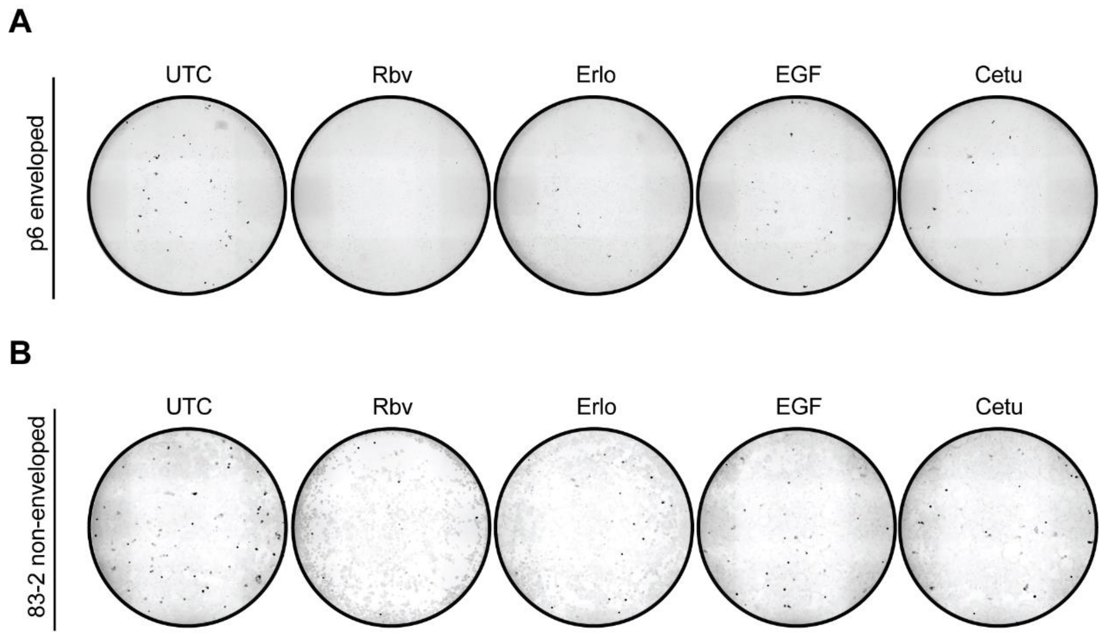

Supplementary Figure S3: Endogenous EGFR is critical for HEV infection. (A) HEVcc p6 enveloped and **(B)** 83-2 non-enveloped infection in HepG2 cells under treatment of EGFR modulators Erlotinib (33 µM, Erlo), EGF (16.5 nM) and Cetuximab (34 nM, Cetu) compared to untreated control cells (UTC), while the HEV inhibitor Ribavirin (50 µM, Rbv) served as control. HEVcc infected cells were stained against ORF2 protein (indicated in black) and images taken using the 4x magnification of the Keyence microscope. Images were stitched and processed using Fiji. Full well images are depicted.

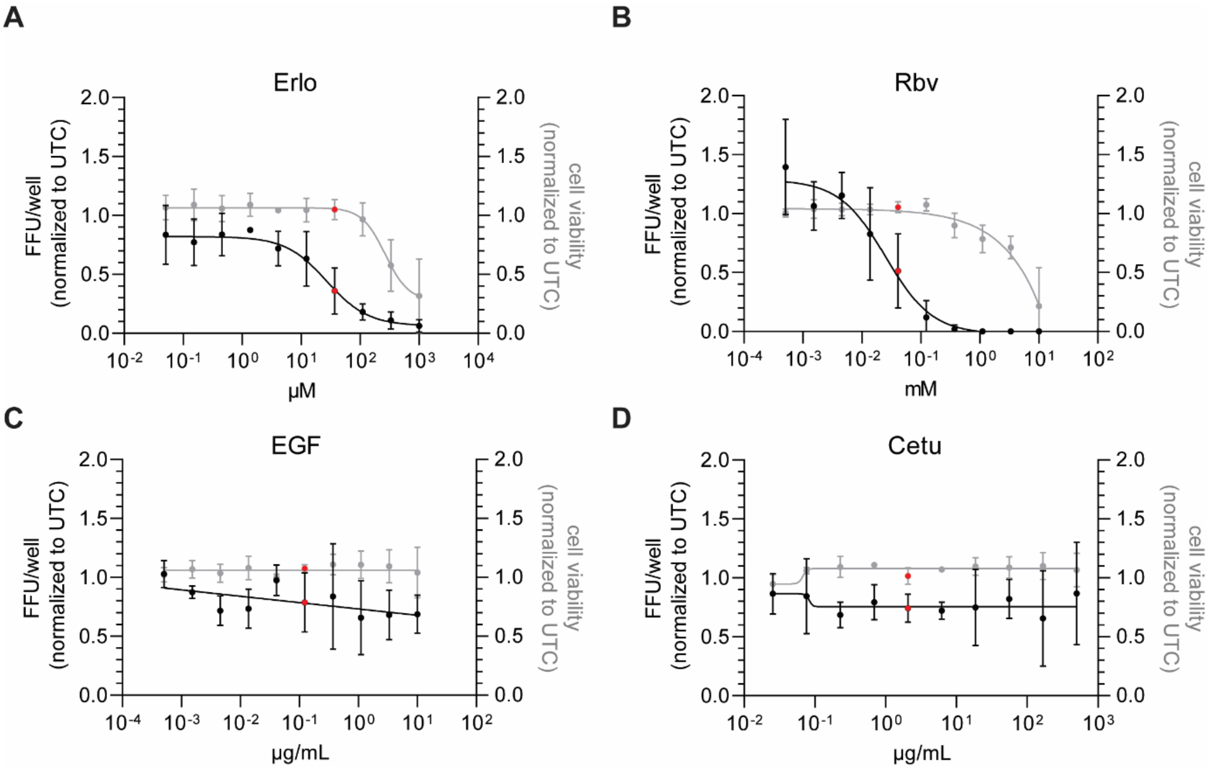

Supplementary Figure S4: EGFR kinase inhibitor Erlotinib inhibits HEVcc p6 infection in a dose-dependent manner. Infection with HEvcc p6 under simultaneous treatment of **(A)** Erlotinib (Erlo) **(B)** Ribavirin (Rbv) **(C)** EGF and **(D)** Cetuximab (Cetu) with the indicated concentrations fixed at 3 d p.i.. FFU/wells (black) normalized to untreated cells. Cell viability (grey) measured using an MTT Assays at 3 days post treatment and normalized to untreated cells. Indicated in red is the concentration used in subsequent infection assays with the specific modulator. All experiments were performed in triplicates. Mean and SEM are depicted from three independent experiments. Dose-dependent treatment was plotted and adjusted to a non-linear regression model.

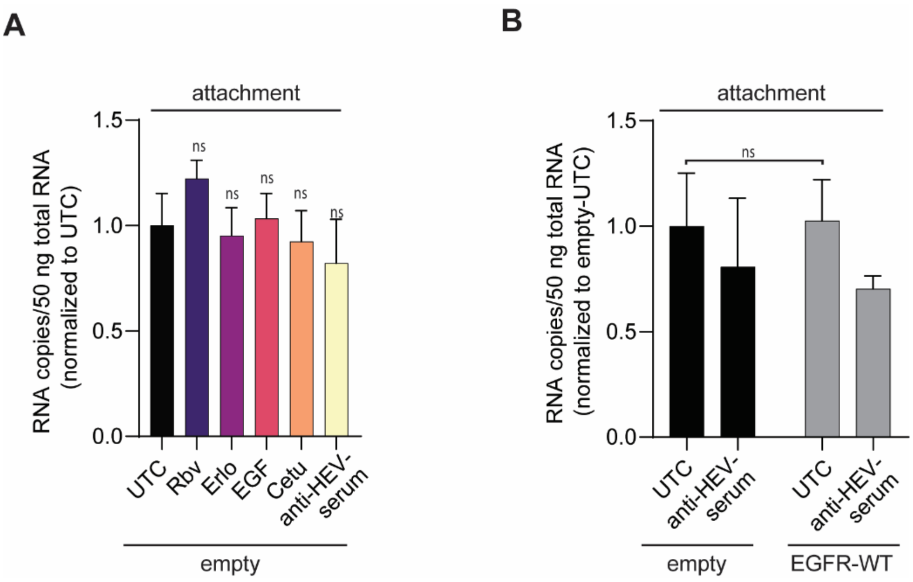

Supplementary Figure S5: EGFR does not affect HEV attachment analyzed via qPCR. Quantification of HEVcc (p6) attachment under EGFR modulator treatment RNA copies/50 ng total RNA isolated and normalized to untreated, infected HepG2/empty cells. **(A)** HepG2-empty cells were pretreated with EGFR modulators for 30 min at 37 °C before addition of virus for 2 h on ice, allowing attachment but not entry. HEV inhibitor Rbv served as negative control here and anti-HEV serum (1:200) as positive control neutralizing HEVcc (p6). Cells were washed thrice before RNA isolation and quantification of HEV RNA copies. **(B)** Either HepG2-empty cells or EGFR-WT ectopically expressing HepG2 cells were pretreated with anti-HEV serum for 30 min at 37 °C before addition of virus for 2 h on ice. Cells were washed thrice before RNA isolation and quantification of HEV RNA copies. To test significance of mean differences, student t-test (B) and one-way ANOVA followed by Dunnett's multiple comparison test (A) were used, p values < 0.05 (*), <0.01 (**), <0.001 (***) and <0.0001 (****), p values >0.05 were considered to be non-significant (ns). All experiments were performed in duplicates. Mean and SEM are depicted from three independent experiments.

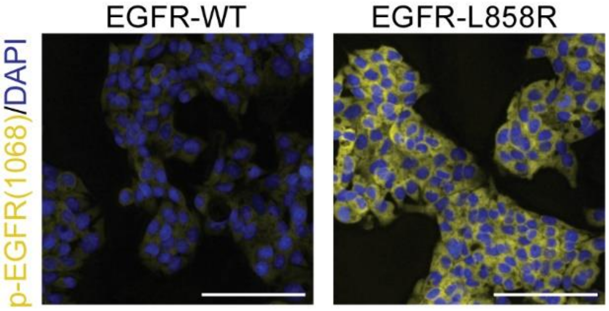

Supplementary Figure S6: EGFR-L858R Mutant is constitutively active. Immunofluorescence analysis of EGFR phosphorylation at Tyr1068 after overnight FCS starvation of EGFR-WT and EGFR.L858R ectopically expressing HepG2 cells. Scalebars = 100 µm.

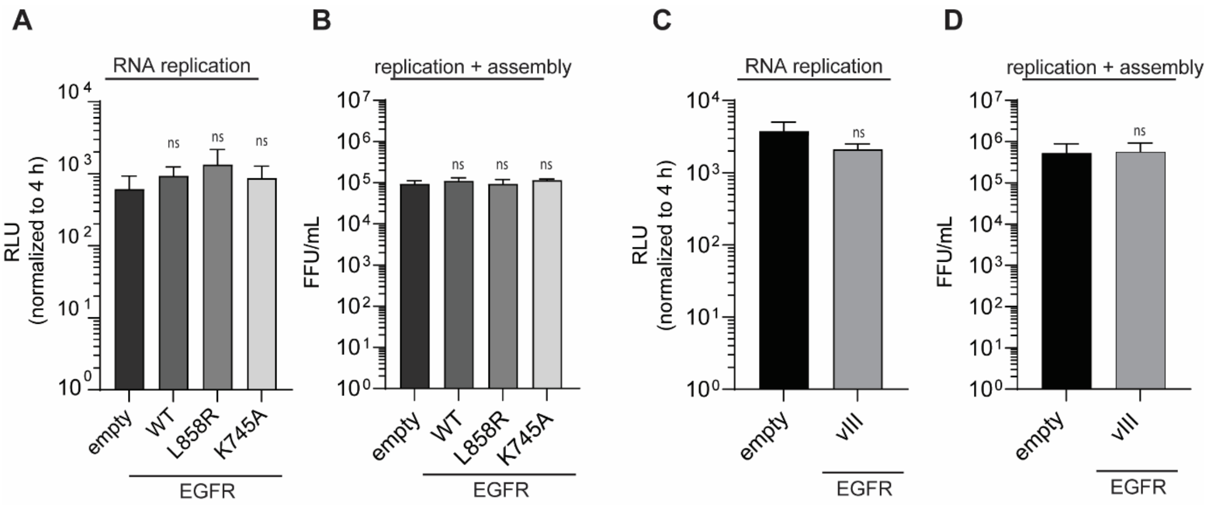

Supplementary Figure S7: Mutant EGFR expression does not affect HEV RNA replication nor production of progeny virus. (A, C) HEV (p6) replication level in RNA subgenomic replicon (SGR) system 72 h.p.e in mutant EGFR ectopically expressing HepG2 cells normalized to relative light unit (RLU) levels at 4 h.p.e.. **(B, D)** Mutant EGFR ectopically expressing HepG2 cells transfected with HEV Kernow-p6 RNA for virus production. Virus titers determined from non-enveloped virus produced in mutant EGFR ectopically expressing HepG2 cells. To test significance of mean differences, student t-test (C and D) and one-way ANOVA followed by Dunnett's multiple comparison test (A and B) were used, p values < 0.05 (*), <0.01 (**), <0.001 (***) and <0.0001 (****), p values >0.05 were considered to be non-significant (ns). Replication experiments were performed in triplicates (A and C), determination of viral titers was performed in duplicates (B and D). Mean and SEM are depicted from three independent experiments.

References

1. Wu X, Dao Thi VL, Liu P, Takacs CN, Xiang K, Andrus L, et al. Pan-Genotype Hepatitis E Virus Replication in Stem Cell-Derived Hepatocellular Systems. Gastroenterology 2018;154:663-674.e7; PMID:29277559; https://doi.org/10.1053/j.gastro.2017.10.041.
2. Rothenberg SM, Engelman JA, Le S, Riese DJ, Haber DA, Settleman J. Modeling oncogene addiction using RNA interference. Proceedings of the National Academy of Sciences of the United States of America 2008;105:12480–12484; PMID:18711136; https://doi.org/10.1073/pnas.0803217105.
3. Zhang C, Freistaedter A, Schmelas C, Gunkel M, Dao Thi VL, Grimm D. An RNA Interference/Adeno-Associated Virus Vector-Based Combinatorial Gene Therapy Approach Against Hepatitis E Virus. Hepatology communications 2022;6:878–888; PMID:34719133; https://doi.org/10.1002/hep4.1842.
4. Todt D, Friesland M, Moeller N, Praditya D, Kinast V, Brüggemann Y, et al. Robust hepatitis E virus infection and transcriptional response in human hepatocytes. Proceedings of the National Academy of Sciences of the United States of America 2020;117:1731–1741; PMID:31896581; https://doi.org/10.1073/pnas.1912307117.
5. Todt D, François C, Anggakusuma, Behrendt P, Engelmann M, Knegendorf L, et al. Antiviral Activities of Different Interferon Types and Subtypes against Hepatitis E Virus Replication. Antimicrobial agents and chemotherapy 2016;60:2132–2139; PMID:26787701; https://doi.org/10.1128/AAC.02427-15.
6. Jothikumar N, Cromeans TL, Robertson BH, Meng XJ, Hill VR. A broadly reactive one-step real-time RT-PCR assay for rapid and sensitive detection of hepatitis E virus. Journal of virological methods 2006;131:65–71; PMID:16125257; https://doi.org/10.1016/j.jviromet.2005.07.004.
7. Behrendt P, Friesland M, Wißmann J-E, Kinast V, Stahl Y, Praditya D, et al. Hepatitis E virus is highly resistant to alcohol-based disinfectants. Journal of hepatology 2022; PMID:35085595; https://doi.org/10.1016/j.jhep.2022.01.006.
8. Aizarani N, Saviano A, Sagar, Mailly L, Durand S, Herman JS, et al. A human liver cell atlas reveals heterogeneity and epithelial progenitors. Nature 2019;572:199–204; PMID:31292543; https://doi.org/10.1038/s41586-019-1373-2.
9. Schindelin J, Arganda-Carreras I, Frise E, Kaynig V, Longair M, Pietzsch T, et al. Fiji: an open-source platform for biological-image analysis. Nature methods 2012;9:676–682; PMID:22743772; https://doi.org/10.1038/nmeth.2019.
